# NLP-based tools for localization of the epileptogenic zone in patients with drug-resistant focal epilepsy

**DOI:** 10.1038/s41598-024-51846-6

**Published:** 2024-01-29

**Authors:** Sara Mora, Rosanna Turrisi, Lorenzo Chiarella, Alessandro Consales, Laura Tassi, Roberto Mai, Lino Nobili, Annalisa Barla, Gabriele Arnulfo

**Affiliations:** 1 Department of Informatics, Bioengineering, Robotics and System Engineering (DIBRIS), University of Genoa, 16145 Genoa, Italy; 2 MaLGa Machine Learning Genoa Center, University of Genoa, 16146 Genoa, Italy; 3 Department of Neuroscience, Rehabilitation, Ophthalmology, Genetics, Child and Maternal Health (DINOGMI), University of Genoa, 16132 Genoa, Italy; 4 Child Neuropsychiatry Unit, IRCCS Istituto Giannina Gaslini, Member of the European Reference Network EpiCARE, 16147 Genoa, Italy; 5 Division of Neurosurgery, IRCCS Istituto Giannina Gaslini, 16147 Genoa, Italy; 6 “Claudio Munari” Epilepsy Surgery Center, Niguarda Hospital, 20162 Milan, Italy; 7 Neuroscience Center, Helsinki Institute of Life Science (HiLife), University of Helsinki, 00014 Helsinki, Finland

**Keywords:** Neuroscience, Biomedical engineering, Epilepsy, Computer science

## Abstract

Epilepsy surgery is an option for people with focal onset drug-resistant (DR) seizures but a delayed or incorrect diagnosis of epileptogenic zone (EZ) location limits its efficacy. Seizure semiological manifestations and their chronological appearance contain valuable information on the putative EZ location but their interpretation relies on extensive experience. The aim of our work is to support the localization of EZ in DR patients automatically analyzing the semiological description of seizures contained in video-EEG reports. Our sample is composed of 536 descriptions of seizures extracted from Electronic Medical Records of 122 patients. We devised numerical representations of anamnestic records and seizures descriptions, exploiting Natural Language Processing (NLP) techniques, and used them to feed Machine Learning (ML) models. We performed three binary classification tasks: localizing the EZ in the right or left hemisphere, temporal or extra-temporal, and frontal or posterior regions. Our computational pipeline reached performances above 70% in all tasks. These results show that NLP-based numerical representation combined with ML-based classification models may help in localizing the origin of the seizures relying only on seizures-related semiological text data alone. Accurate early recognition of EZ could enable a more appropriate patient management and a faster access to epilepsy surgery to potential candidates.

## Introduction

Epilepsy is a neurological disorder characterized by recurrent seizures, which are abnormal electrical discharges in the brain. Focal-onset seizures, also known as partial seizures, begin in a specific region of the brain, as opposed to generalized seizures that affect the entire brain. When epilepsy is referred to as drug-resistant (DR) or refractory, it means that seizures persist despite adequate trials of antiepileptic drugs. Focal-onset drug-resistant epilepsy can potentially be resolved through surgery. However, this option remains underutilized due to several factors, including patient misconceptions about the procedure, economic disparities in healthcare access, and the complexity of identifying suitable candidates. A critical challenge lies in the difficulty of pre-surgical assessment, which entails classifying the type of seizure, localizing and lateralizing the Epileptogenic Zone (EZ), and assessing the safety of the intended surgical procedure in consideration of potential deficits (motor, cognitive, etc.)^1^. Long-term Video-electroencephalography (VEEG) monitoring is a diagnostic technique commonly used to objectively capture both clinical manifestations and brain activity during seizures^2^. Accurate interpretation of both subjective and objective manifestations related to seizures is paramount for developing a robust hypothesis about the potential location of the epileptogenic zone (EZ).

Typically, epileptologists meticulously review numerous seizure manifestations obtained from long-term VEEG recordings and provide a comprehensive report detailing the characteristics of the semiological manifestations (e.g., motor/non-motor) and their chronological appearance^3^. Subsequently, an hypothesis concerning the location of the EZ is formulated, guiding the planning of surgical interventions when corroborated by electroencephalography (EEG), Magnetic Resonance Imaging (MRI), or other functional data. Alternatively, this hypothesis may steer additional pre-surgical evaluation phases, including invasive procedures. Given the intricate nature of this process, it necessitates specific skills acquired through years of experience^4^.

An innovative approach consists in leveraging Machine Learning (ML) models to build automatic decision support systems capable of achieving high accuracy in the interpretation of clinical data^5–9^ as well as offering support in formulating optimal therapeutic options^10^. ML-based tools can be employed to automatically analyze clinical reports detailing seizure manifestations, providing an important tool to support clinical diagnosis of people with DR epilepsy. However, it is important to note that the collected reports, typically in text-based unstructured formats, pose a challenge for straightforward ML analysis alone. In this context, Natural Language Processing (NLP) emerges as a potentially disruptive methodology. Indeed, NLP is the branch of Artificial Intelligence focusing on the computerized analysis of natural human language. The applications of NLP in the clinical field are diverse^5,6,11^ and, when combined with ML methods, they can significantly contribute to the diagnostic process.

The growing interest in the synergistic use of ML and NLP techniques within the epilepsy field has already spurred several research projects, particularly focusing on application in supporting differential diagnosis and management in epilepsy syndromes^12–14^. While some efforts have been made in predicting the localization of EZ, these approaches often depend on expert clinicians identifying meaningful keywords, either manually or through regular expressions^15,16^. Further, these studies are constrained by predefined rules, requiring additional efforts for widespread adoption, and are potentially biased as they rely solely on clinicians’ experiences. Consequently, the automatic analysis of semiological descriptions for EZ location remains an open question^17^.

This paper introduces a computational pipeline that integrates a ML-based classification with NLP models capable of lateralizing (right or left) and localizing (temporal or extra-temporal, frontal or posterior) the EZ, relying solely on the text-based semiological descriptions of seizures.

## Methods

### Inclusion criteria

We conducted a retrospective review of clinical reports of subjects diagnosed with epilepsy at the “Claudio Munari” Epilepsy Surgery Centre, Niguarda Hospital in Milan (Italy). From this pool, we selected patients with focal DR epilepsy who attained seizure freedom post-surgical intervention, ensuring a minimum follow-up period of two years. This meticulous approach allowed for the precise identification of the the origin of seizures, namely the epileptogenic zone. The resulting cohort comprises 127 patients. The summary of the localization and hemisphere of the EZ distributions are summarized in Table 1, with the exception of two patients who lacked one or both pieces of necessary information. Detailed information for each patient about epilepsy symptoms, etiology, precise location of EZ, and surgical resection can be found in Supplementary Table 2.

All participants gave informed consent for data collection and usage for scientific research (ID 939-12.12.2013). This is an anonymous retrospective study that complies with the principles outlined in the Declaration of Helsinki^18^.

**Table 1:** Number of patients for each combination of localization and side.

Localization	Side	Patients (*n*)
Frontal	Right	14
	Left	17
Temporal	Right	32
	Left	30
Central	Right	2
	Left	1
Insulo-opercular	Right	5
	Left	4
Posterior	Right	11
	Left	9
Hemispheric	Right	1
	Left	1

### Samples characteristics

For each patient, we gathered information on the localization and lateralization of the EZ and two sets of textual data written in Italian, one comprising descriptions of all available seizures and the other containing excerpts from the patients’ Electronic Medical Records. Specifically:

*Seizure descriptions* are texts describing the semiology of seizures. In particular, medical experts revised recorded videos capturing patients during seizure events, providing comprehensive description of the manifestations and evolution of each seizure. We examined all seizure descriptions (N = 566) excluding those that: (i) referred to previous seizures (e.g., the sentences like *“Seizure similar to the previous ones including the automatisms of the right hand brought to the face”*); (ii) comprised fewer than 20 words (following the data cleaning phase outlined in the upcoming section “Data pre-processing”). After this refinement, 30 texts were excluded, resulting in a dataset of 536 descriptions from 122 patients out of the initially included 127. The average number of seizures per patient is $4.39 \pm 3.63$, ranging from 1 to 17. We treated single seizure descriptions as independent events as ictal events occurred at different times, involving different clinicians documenting the semiological description.

*De-identified excerpt of Electronic Medical Records (EMRs)* containing anamnestic information of 127 patients such as patient’s history, previous treatments, drug-dosage, etc.

In order to preserve the morphological structure of the sentences and ensure de-identification during pre-processing, we removed Protected Health Information (PHI). This step constituted the sole text manipulation, performed manually. The de-identified texts were then stored in a SQL Server database located in a server accessible only through a Virtual Private Network. Each patient was assigned a unique identifier, and we retained only minimal personal information, such as sex and year of birth, adhering to international and national regulations on data protection^19,20^.

Further, expert epileptologists assigned two types of labels for patient: the location (i.e., the region) and the side (i.e., right/left) of the brain where the EZ is situated. This information is available as all patients underwent surgical intervention that resolved the pathological condition. The first label type distinguishes whether the seizure originates from the temporal ($n_{temporal}=59$) or extra-temporal ($n_{extra-temporal}=63$) brain region. The extra-temporal label includes patients whose EZ either does not exclusively cover the temporal region or has an extra-temporal location (frontal, parietal, etc). Further, for patients (49) with extra-temporal epilepsy, experts provided information about whether the seizure onset site is frontal ($n_{frontal}=29$) or posterior ($n_{posterior}=20$). The second label type categorizes the EZ based on the hemisphere in which it is located in (right ($n_{right}=62$) or left ($n_{left}=60$)). Considering that, as previously mentioned, each patient may experience more than one seizure, the dataset is composed of 58% of seizures labeled in the extra-temporal region, 64% of which are labeled as frontal, and 57% of seizures associated to the right hemisphere.

**Figure 1:**
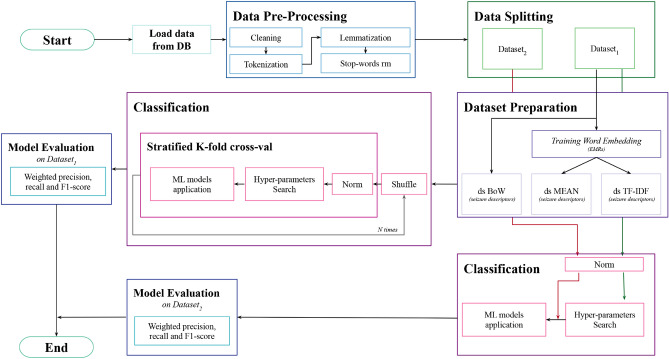
Pipeline Schema. The pipeline, implemented in Python 3 language, can be divided into 5 main sections: data pre-processing, data splitting, dataset preparation, classification and model evaluation.

The objective of our study is to build predictive models based only on seizure descriptions represented according to a specific embedding criterion. The problem is cast into a supervised learning framework, where each seizure is associated to a label (temporal or extra-temporal, frontal or posterior, left or right). The entire experimental pipeline encompasses five phases (data pre-processing, data splitting, dataset preparation, classification and model evaluation), outlined in the subsequent sections. The comprehensive schema of the pipeline is illustrated in Fig. 1.

### Data pre-processing

The data processing phase is composed of four steps described in the following.

*Data cleaning* Patterns containing numbers, e.g., dates or names of electrodes, punctuation, text in brackets^21^ were removed from the sentences by using regular expressions though ‘re’ Python module (https://docs.python.it/html/lib/module-re.html). Further, we extended common abbreviations used by clinicians in their daily practice, e.g., *“aass”* which means upper limbs and *“aoo”* which means eyes open.

*Tokenization* The content of the text was split into minimum units of analysis (tokens)^22^, e.g., single words or groups of specific words, using the *‘Natural Language ToolKit’ library*^23^.

*Lemmatization* Each word was assigned to its base form (lemma, e.g., verbs were turned to infinite form and plurals became singular) using ‘TreeTaggerWrapper’ (https://treetaggerwrapper.readthedocs.io/en/latest/) to uniform the text (normalization process)^24^.

*Stop-words removal* Stop-words, that include common words like articles and prepositions that lack informativeness and may interfere with model construction, were removed from the text. The complete list is available in ‘spaCy’ module (https://spacy.io/models/it).

An illustrative example of text both before and after the automatic manipulation can be found in the supplementary materials.

### Data splitting

After the pre-processing phase, the dataset was randomly split into two sets: *Dataset*_1_ comprising 464 seizures from 107 patients and *Dataset*_2_ consisting of 72 seizures from 15 patients. The division is meant to assess the generalization of the representation model, as well as the predictive one. Indeed, as further detailed in the following section, the representation construction and the training process exclusively leverage *Dataset*_1_ (or a subset of it), while *Dataset*_2_ is solely employed for testing purposes. It is crucial to note that for the frontal versus posterior classification task, only the subset of patients with extra-temporal epilepsy is considered both in *Dataset*_1_ and *Dataset*_2_. Specifically, we selected 222 seizure descriptions related to 44 patients in *Dataset*_1_, and 26 seizure description corresponding to 5 patients in *Dataset*_2_.

### Data preparation: NLP and text representation

To construct a quantitative and meaningful representation of the seizures descriptions, suitable for input into learning algorithms, we transformed them from textual data into three distinct numerical matrices. This was achieved using two text representation methods: Bag of Words and Word Embedding.

#### Bag of words

A standard sparse representation of the text, discarding the order of words and capturing the frequency of patterns within a document. More precisely, these patterns are *n*-grams of tokens, which are sequences of n items (characters and/or words) within a document^25^. Typically, only the most frequent *n*-grams of tokens are considered, excluding less common ones. The resulting numerical representation has a shape of number of samples $\times$ number of features, where the latter corresponds to the number of *n*-grams of tokens. To build the text representation, all 464 available seizures descriptions in *Dataset*_1_ were utilized. We employed both *n*-grams of characters (with $n=2,3$) and *n*-grams of words (with *n* from 1 to 4), in the proportion of 20% and 80% respectively, and retaining only the most frequent ones within each group. *n*-grams of characters were used to address misspellings, while *n*-grams of words preserved some contextual information that would otherwise be lost, given that this technique discards information about the order of tokens^26^. The extraction of the *n*-grams of tokens was carried out solely from the seizure descriptions of the patients in the learning set (*Dataset*_1_). To create this numerical representation, we utilized ‘CountVectorizer’ (https://scikit-learn.org/stable/modules/generated/sklearn.feature_extraction.text.CountVectorizer.html) from ‘Scikit-learn’^27^. To determine the optimal size for the numerical representation, we tested three choices for the total number of features: 100, 200, and 300. We obtained best results with 200 features in the first task and with 300 features in the second task. From now on we will refer to this numerical representation with *bw*.

#### Word embedding

A dense numerical representation of words in a continuous vector space, where semantically similar words are mapped to nearby points. Unlike traditional methods that represent words as discrete symbols or indices, word embeddings capture semantic relationships and context. This approach is based on deep learning models mapping a word *w*, from a vocabulary *V*, to a real-valued vector in an embedding space of dimension *D*. In our experiments, we adopted *Word2vec*^28^ as deep learning model as demonstrated that it excels in generating word embeddings for a wide range of general NLP tasks compared to other approaches^29,30^.

One of the main differences between these two representation methods is that the Bag of Words approach provides a direct representation of the entire document, whereas the Word Embedding model operates at the word level. Consequently, when utilizing Word Embedding, we conducted a preliminary analysis of the quality of the word representations before constructing the overall document representation. As suggested in^30,31^, we employed the following intrinsic evaluators.

*Words similarity* It is defined as

1 $$\begin{aligned} \cos (w_1,w_2) = \frac{w_1 *w_2}{\mid \mid w_1 \mid \mid *\mid \mid w_2 \mid \mid }, \end{aligned}$$

where $w_1$ and $w_2$ are the two word vectors and $\mid \mid w_1 \mid \mid \text {and} \mid \mid w_2 \mid \mid \text {are} \ L_2$ norms.

*Words analogy* Given a pair of related words (*a* and $a^*$) and a third word (*b*), the analogy relationship between *a* and $a^*$ can be used to find the word $b^*$ that corresponds to *b*, such as

2 $$\begin{aligned} a :a^*= b :b^*. \end{aligned}$$

*Outliers detection* Given a group of words, the objective is to find the one that does not match the context and therefore to evaluate the semantic coherence in words clusters.

Given that a substantial amount of text is required to train the Word2vec model, we used both the set EMRs along with the 464 seizure descriptions in *Dataset*_1_. Further, the use of EMRs may enhance the resulting embedding as these texts often comprise syntactically complete sentences, providing valuable in capturing relationship between words.

From the EMRs, we excluded text sections containing clinical conclusions related to the EZ location, to prevent potential influence on the relationship among words vectors in the embedding space. Various vector dimensions and combinations of parameters of the *Word2vec* model were investigated, following reference range outlined in^32^. We identified the optimal values through intrinsic evaluators, which are: vector dimension=100; negative sampling = 10 and number of epochs = 300. Then, we set the minimum words occurrence in the text to 2, in order to exclude overly rare words or misspellings of frequent words, and number of context words equal to 3. Finally, we derived the representation of the entire document representation following two different approaches:

*mean representation*, in which we averaged all the word representations.

*tfidf representation*, in which we applied the *Term Frequency-Inverse Document Frequency* (TF-IDF) formula to words vectors^33^.

In total we obtained 3 text representations per dataset: the first one using the Bag of Words (*bw* representation) and the other two based on the Word Embedding model (*mean* and *tfidf* representations).

### Classification: ML methods

For each input representation, we cast a binary classification problem for three different tasks. Two tasks aim at predicting the brain region of the seizure onset: the first one discriminates between temporal and extra-temporal sites, while the second one classifies the patients within extra-temporal group into subjects with frontal or posterior seizure onset sites. Note that the latter task is more challenging as it only uses a subset of $Dataset_1$ of 222 seizure descriptions, related to 44 patients with extra-temporal epilepsy. Finally, the third task predicts the brain hemisphere (left/right) where seizures originate. We adopted and compared two different ML classification methods, that are Sparse Logistic Regression with $L_1$ penalty^34,35^ and Support Vector Machine (SVM)^36,37^ with three different kernel function: linear, radial basis function (rbf), polyonimial with degree equal to 3 (poly). Hence, in total we had 4 models per each input and task.

For all experiments, we performed a stratified k-fold cross-validation, with k=10, to iteratively split *Dataset*_1_ into ten different training and testing sets. At each split, the following steps were performed:

1. Data normalization
2. Best hyperparameters search via 10-fold cross-validation on the training set
3. Model training on the training set for fixed optimal hyperparameters
4. Model evaluation on the testing set

All the aforementioned steps have been executed $N=3$ times, with data shuffled each time. To ensure result reproducibility, we set the random state used for data shuffling equal to the iteration index (i.e., in order 0, 1, 2). Note that *Dataset*_2_ has not been employed during this phase.

### Model evaluation

The overall performance of each model over multiple trials was computed calculating the median performance per trial and the mean performance across the $N=3$ trials. All experiments have been evaluated based the following weighted metrics for each fold and on average: accuracy (i.e., percentage of correct predictions), precision (i.e., positive predictive value, where positive classes are ‘left’, ‘extra-temporal’, and ‘posterior’), Negative Predictive Value (NPV; negative classes are ‘right’, ‘frontal’ and ‘temporal’), specificity, and F1-score^38–40^.

### Ethics approval statement

This is an anonymous retrospective study that complies with the principles outlined in the Declaration of Helsinki^18^. The current study received the approval of the Niguarda Hospital ethics committee (ID 939-12.12.2013).

### Patient consent statement

All participants gave informed consent for data collection and usage for scientific research.

## Results

### Evaluation of the Word Embedding

Before constructing the numerical representations of seizure descriptions, we assessed the Word2Vec model performance using three different evaluators to evaluate the word representation.

Firstly, we tested if the Word Embedding correctly recognized the semantic and syntactic meaning of random words. Five target words were chosen, and the most similar words extracted from the Word Embedding based on the Word Similarity measure defined by Eq. (1). Our model accurately associated words with syntactic and semantic meanings similar to the target words in all the selected cases (see Table 2). For instance, the word “sollevamento” *(lift)* is one of the most similar words to “movimento” *(movement)* but it is also a very similar to “elevazione” *(elevation)* and this semantic proximity is recognized by the model (Suppl. Fig. S1).

Secondly, we assessed our model using the Words Analogy evaluator. We aimed to find the word satisfying the following relation: “braccio” *(arm)* + “gamba” *(leg)* − “piede” *(foot)*. We correctly obtained the word “mano” *(hand)*.

Finally, we evaluated the model’s ability to detect outliers, evaluating if it could recognize words out of their typical context. Specifically, we selected a quadruplet of words: three within the same context and one outlier. This experiment was repeated three times, consistently demonstrating that our model successfully detect the outlier (see Table 3). Overall, these results indicate that the designed and trained model properly identifies relations between words.

**Table 2:** Illustration of words similarity evaluation.

Target word	Most similar words
Braccio *(arm)*	Gamba, mano, sinistro, destro, superiore *(leg, hand, left, right, upper)*
Episodio *(episode)*	Crisi, soggettivamente, risveglio, critico, manifestazione *(seizure, subjectively, awakening, critical, manifestation)*
Clonia *(jerk)*	Mioclonia, scossetta, disordinare, elevazione, sollevato *(myoclonia, jerk, clutter, elevation, raised)*
Scuola *(school)*	Ragioneria, liceo, elementari, scolarità, impiegare *(accounting, high school, primary school, schooling, employ)*
Movimento *(movement)*	Muovere, sinistro, destro, capo, sollevamento *(move, left, right, head, lift)*

The first column showcases five target words selected arbitrarily, while the second column presents the corresponding five most similar words generated by the Word Embedding model. Most similar words are reported in descending order, based on the cosine similarity coefficient.

**Table 3:** Examples of the outliers detection evaluator.

Input words	Outliers
Dormire, cuscino, letto, bicchiere *(sleep, pillow, bed, glass)*	Bicchiere *(glass)*
Scuola, braccio, gamba, mano *(school, arm, leg, hand)*	Scuola *(school)*
Fratello, sorella, madre, crisi *(brother, sister, mother, seizure)*	Crisi *(seizure)*

The left column reports quadruplets of words, whereas the right column shows the identified outlier.

### Left versus right hemisphere seizure onset sites

**Figure 2:**
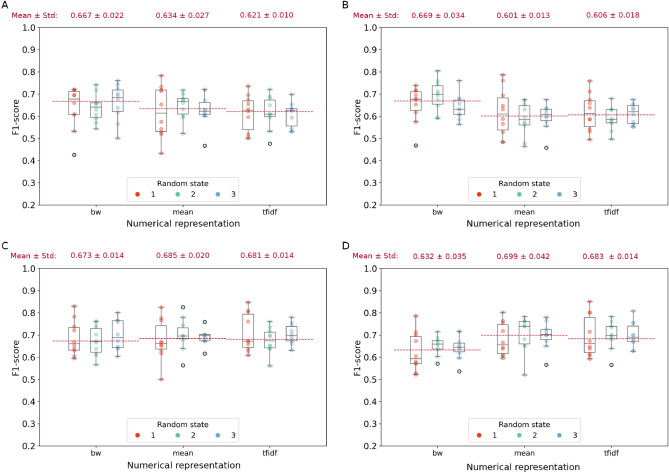
Weighted F1-scores of classification model for the Right versus Left lateralization task obtained on testing set of (**A**) Sparse Logistic Regression, (**B**) SVM with linear kernel, (**C**) SVM with rbf kernel, and (**D**) SVM with polynomial kernel over the three fixed random states (red, light green, and light blue) and the three numerical representations (*bw*, *mean*, and *tfidf*). For each representation and random state, the weighted F1-score values of the k-folds are showed. The red dotted lines identify the mean of second quartiles over the three random states. Numbers at the top of each panel represent $\mu \pm \sigma$ of the second quartiles over the three random states.

The first learning task consisted of a predictive model able to determine the lateralization (*i.e.*, left versus right hemisphere) of the EZ. Sparse Logistic Regression and linear SVM reached the highest F1-score values when using *bw* representation, whereas SVM with rbf and polynomial kernel showed better performances using Word Embedding-based representations, as shown in Fig. 2. The SVM with rbf kernel with *mean* representation yielded the best performance overall with an F1-score of $68.5\% \pm 2\%$. Accuracy, precision, NPV, and specificity for each combination of models and word representation can be found in Supplementary Materials, in Fig. S4. Observing the confusion matrices in Fig. 5, the low overall performances are mostly due to the large number of seizures classified as right origin while being originating from the left hemisphere. Our results confirmed that predicting lateralization of seizure onset represents a complex task based solely on semiology descriptions.

### Temporal versus extra-temporal seizure onset sites

**Figure 3:**
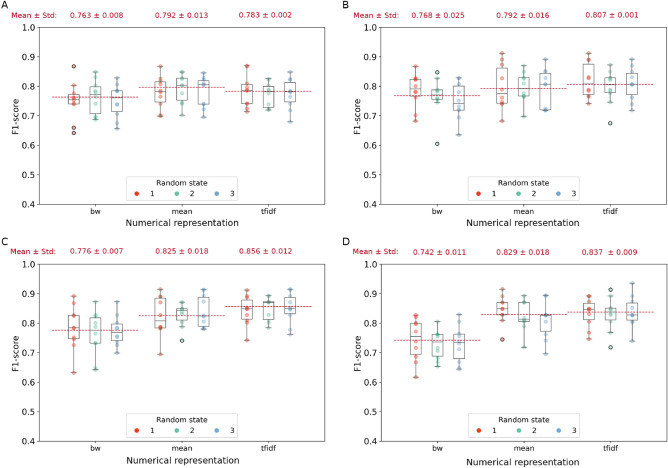
Weighted F1-scores for the localization task on testing set of (**A**) Sparse Logistic Regression, (**B**) SVM with linear kernel, (**C**) SVM with rbf kernel, and (**D**) SVM with polynomial kernel over the three fixed random states (red, light green, and light blue) and the three numerical representations (*bw*, *mean*, and *tfidf*). For each representation and random state, the weighted F1-score values of the k-folds are showed. The red dotted lines identify the mean of second quartiles over the three random states. Numbers at the top of each panel represent $\mu \pm \sigma$ of the second quartiles over the three random states.

In the second classification task, we aimed at predicting the temporal or extra-temporal origin for a given seizure. Temporal lobe epilepies tend to have a more representative clinical manifestations^41^ leading to a more accurate diagnosis. Overall results on *Dataset*_1_ demonstrated that the models using Word Embedding-based representations outperformed those based on Bag of Words considering all metrics but NPV and sensitivity with values above 80% (Figs. 3 and S2). According to the F1-score measure, Sparse Logistic Regression reached the highest performance using *mean* representation, whereas SVM with *tfidf* representation outperformed the other methods independently of the kernel choice. Among all possible combinations, the use of SVM with rbf kernel and *tfidf* representation provided the best classification performance, identifying the EZ location with an F1-score of $85.6\% \pm 1.2\%$. Confusion matrices confirmed the observation from F1-score and further consolidated the results showing that both temporal and extra-temporal classes were correctly classified, see Fig. 5.

To assess the generalization of both data representation and classification models, we further tested our pipeline on *Dataset*_2_. The best combination of model and word representation obtained performances above 79% in terms of F1-score. Specifically, Logistic Regression reached an F1-score of 72.41% with *mean* representation, 70.78% with *tfidf* representation and 79.15% with bw. SVM with linear kernel reported an F1-score of 75.84%, 73.00% and 77.64% using *mean*, *tfidf* and *bw* representations, respectively.

SVM with rbf kernel obtained an F1-score of 68.58% with *mean*, 71.44% with *tfidf*, and 78.84% with *bw*. Finally, SVM with the polynomial kernel reached F1-scores equal to 73.38% for *mean*, 72.16% for *tfidf*, and 70.38% for *bw*. Further details about accuracy, precision, NPV, and specificity for each combination of model and word representation can be found in Supplementary Materials, in Table S1.

In conclusion, the devised models were able to accurately classify seizure onset location based on semiology notes. Although this was a binary classification task with unbalanced classes, the best model was able to correctly separate each class.

### Frontal versus posterior seizure onset sites

**Figure 4:**
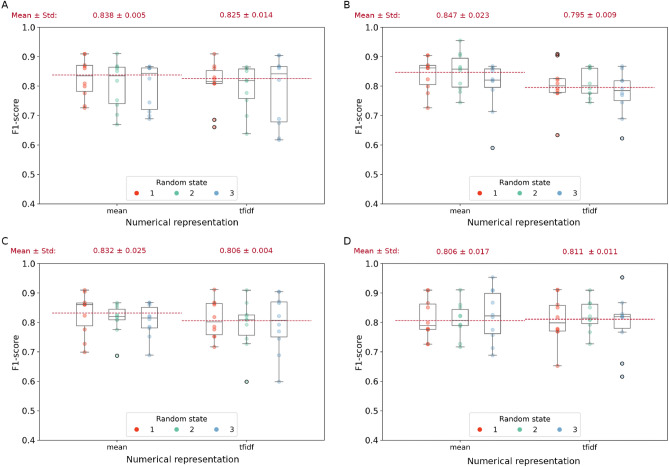
**Weighted F1-scores for the localization in frontal versus posterior region task on testing set** of (**A**) Sparse Logistic Regression, (**B**) SVM with linear kernel, (**C**) SVM with rbf kernel, and (**D**) SVM with polynomial kernel over the three fixed random states (red, light green, and light blue) and the three numerical representations (*bw*, *mean*, and *tfidf*). For each representation and random state, the weighted F1-score values of the k-folds are showed. The red dotted lines identify the mean of second quartiles over the three random states. Numbers at the top of each panel represent $\mu \pm \sigma$ of the second quartiles over the three random states.

**Figure 5:**
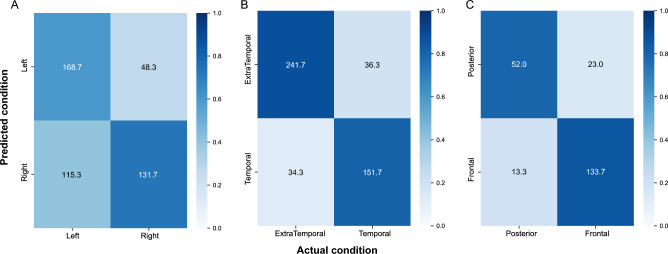
**Confusion matrices for the classification tasks** in (**A**) right versus left, (**B**) temporal versus extra-temporal, and (**C**) frontal versus posterior origin for the task. True Positive are shown in top-left corner, True negative in bottom-right corner, False negative is bottom-left, and False positive in top-right corner. Numbers are the total number of seizures assigned to each category, summed across folds and averaged across repetitions. Color intensity is proportional to the fraction total number of seizures divided by the element in each class.

In the end, we improved the last classification task to better distinguish the seizure onset location in patients with extra-temporal epilepsy, specifically separating between frontal and posterior origin. Seizures from the frontal regions may show clinical manifestation similar to those from the posterior regions, making it challenging to diagnose accurately. Due to the overall lower performance of Bag-of-Words models in previous tasks, we decided to concentrate solely on models using Word-embedding methods for this analysis. Results on extra-temporal epilepsy patients of $Dataset_1$ showed an F1-score always higher than 80% (Fig. 4), where the best score of $84.7\% \pm 2.3\%$ is reached by combining the *mean* numerical representation with SVM with linear kernel. Figure S3 in the Supplementary Materials is where all metric values are reported. Moreover, when testing the generalization’s ability of the best model on $Dataset_2$, we obtained an F1-score of 76.51%. Confusion matrices confirmed the observation from F1-score and further consolidated the results showing that both frontal and posterior classes were correctly classified (Fig. 5).

## Discussion

There is urgent need to accelerate the process that determines whether an epileptic patient is a suitable candidate for surgery or not. Clinical manifestations reported by expert epileptologists while witnessing a seizure and/or during a video-EEG can yield important evidence about the localisation of the epileptogenic zone^41–43^.

Here we proposed a supervised learning model that exploited numerical representation of textual data to automatically localise or lateralise the possible origin of seizures of drug-resistant epileptic patients. Combining methods of shallow learning with different kernels and 3 different word representation techniques, we showed that NLP-powered tools can accurately recognise seizures with different origins. As expected models based on word embedding techniques outperformed those using bag-of-words representation, although the latter seemed more robust towards generalisability. While predicting the hemisphere yielded unsatisfactory results, our analysis demonstrated that temporal from extra-temporal seizures can be separated with sufficient accuracy only relying on semiology notes. Surprisingly, within the extra-temporal class, the best combination of classifier and word representation accurately separated seizures of frontal origin from those of posterior onset. In conclusion, when testing for generalisability the best classificators of each task were able to properly recognise individual classes and achieved performances above 70% on unseen data.

### Localising individual seizures

Reaching an accurate localistion of the epileptogenic zone is the key element to guide surgical decision. The best surgical outcome is usually reached when clinical manifestation in the period of seizure implementation aligns with electrophysiological modifications^44^. Machine learning models are gaining increased interest in medicine and found application in epilepsy^45^ for drug-selection^10^, estimating surgical candidacy^46,47^, and predicting seizure origin^48^. Our models confirmed that seizures with temporal lobe origin were more easily identifiable from those arising from extra-temporal regions with accuracy comparable with previous works^8,48^. Our approach extends the existing models by attempting to localise with finer precision extra-temporal seizures. Performing surgery for epilepsy from the posterior quadrant remains uncommon and has shown limited success, particularly in cases of non-lesional epilepsies. This is attributed to intricate connectivity mechanisms, deceptive semiology, and non-localizing EEG recordings, potentially stemming from insufficient synchronicity in the parietal cortex^49^. Surprisingly, our results showed that seizures from frontal lobe can be differentiated from those of posterior origin with accuracy greater than 80%.

Our analysis indicates that embedding models perform optimally on the learning set (*Dataset*_1_), as also showed in the literature^29^. On unseen data (*Dataset*_2_), the bag-of-words representation also exhibits good performance.

### Lateralisation represents a more challenging task

We also attempted to lateralise seizure onsets based on the semiology descriptions. It should be noted that the lateralization (left vs. right) task presents some additional complexities. Specifically, some clinical signs that possess high lobe-localizing value may lack lateralization value (e.g., epigastric aura in mesial temporal lobe epilepsy), while some clinical signs (e.g., head version) may address ipsilateral or contralateral localization depending on which neuronal network is being activated. Moreover, some focal seizures may occur with bilateral signs, in which the detection of asymmetries with lateralization value is particularly challenging and consequently clinician-dependent (e.g., hypermotor seizures in frontal lobe epilepsy). Finally, some clinical and potentially lateralizing signs such as ictal/postictal aphasia may have not been always tested. Moreover, the lateralizing value of these signs may be relative to hemispheric dominance (dominant vs. non-dominant hemisphere), thus not expressing an exact left-right distinction value.

The main advantage of proposed approach is its independence from epilepsy-specific information, such as ontologies, during the model-building phase. This characteristic makes our pipeline adaptable to various clinical scenarios beyond epilepsy. To the best of our knowledge, this work constitutes the first NLP-based diagnostic tool for drug-resistant focal epilepsy able to provide a classification on potential origin of seizure using only semiology descriptions and designed specifically for Italian centers. The project challenges were amplified by the absence of pre-trained embedding models for biomedical applications in the Italian language, a gap not addressed by existing works on this topic. Our work also presents some limitations. Physician’s writing style and experience in noticing and reporting meaningful event could affect numerical representation. Indeed, the variability in clinicians’ writing styles, including the use of different synonyms, affects the construction of both text representations. This impact is especially evident in training the word embedding model, where each word depends on its context (other nearby words). The most relevant features extracted by the count-based model are also influenced by their frequency, further emphasizing the impact of individual clinicians. It should be however noted that recently it has been proved that NLP-based diagnostic support system could benefit from variability in text representation and that NLP-based systems can be successfully trained on data from centers and provide similar accuracy when tested on data from different centers^50^.

Additionally, the study limitation stems from the relatively low number of patients included, all from the same center. Consequently, the number of seizure descriptions is limited, and text variability is constrained by the fixed number of clinicians working within the institution. To address these limitations, future work aims to extend the study to involve other Italian centers dedicated to epilepsy diagnosis and management.

## Conclusions

In conclusion, identifying the EZ poses a significant challenge in assessing patients with DR focal epilepsy patients. Our findings serve as a foundational step in developing a non-invasive, cost-effective tool. This tool has a the potential to serve as a valuable aid in the pre-surgical evaluation conducted in highly specialized centers and offer support in primary-care units, where various diagnostic procedures may not be readily available. In both scenarios, such a tool could reduce the time between epilepsy onset and surgery, leading to a substantial improvement in patients’ quality of life and a reduction in healthcare expenditures.

### Supplementary Information

Supplementary Information.

Supplementary Table S2.

## Data Availability

The datasets generated and/or analysed during the current study are not publicly available due to the high personal content of the texts. However the Word Embedding trained model and the selected list of features of Bag of Words are available from the corresponding author on reasonable request.
