# Supplementary material for: NLP-based tools for localization of the epileptogenic zone in patients with drug-resistant focal epilepsy
Source: Sci Rep. 2024 Jan 29;14:2349. doi: 10.1038/s41598-024-51846-6 (PMC10825198; doi:10.1038/s41598-024-51846-6)
Supplement: Supplementary file 1 — Supplementary Information. [file 41598_2024_51846_MOESM1_ESM.pdf]

Additional Files

Text pre-processing example

Text before pre-processing phase:
15.53.16: sta per cominciare il tracciato di base con la mano dx che tiene gli oo
26: mette tutte e due le mani sugli occhi
42: la mano sx si solleva dall'occhio sx
43: si solleva ancora di più ed abduce il braccio, forse apre gli occhi
45: dice di avere una altra sensazione
50: rimette le mani sugli oo e
54: nuova abduzione del gomito sx

Text after pre-processing phase:
cominciare tracciato base mano destro tenere occhio mettere mano occhio mano sinistro sollevare occhio sinistro sollevare abdurre braccio aprire occhio sensazione rimettere mano occhio abduzione gomito sinistro

Supplementary tables

Table S1. *Performances on Dataset2 in the brain region identification. For each combination of numerical representation and classifier values of accuracy, precision, NPV, and specificity are reported (weighted recall equals to accuracy).*

Classifier	Numerical representation	Accuracy	Precision	NPV	Specificity
**Sparse Logistic Regression**	*bw*	0.722	0.723	0.733	0.647
	mean	0.736	0.767	0.857	0.529
	tfidf	0.722	0.757	0.850	0.5
**SVM with linear kernel**	*bw*	0.736	0.739	0.759	0.647
	mean	0.764	0.778	0.84	0.618
	tfidf	0.736	0.748	0.8	0.588
**SVM with rbf kernel**	*bw*	0.681	0.682	0.690	0.588
	mean	0.694	0.706	0.75	0.529
	tfidf	0.722	0.736	0.792	0.559
**SVM with poly kernel**	*bw*	0.750	0.760	0.808	0.618
	mean	0.736	0.739	0.759	0.647
	tfidf	0.722	0.722	0.719	0.676

Table S2. *Short description of patients' epilepsy.*

SupplTable2.xlsx

Supplementary figures

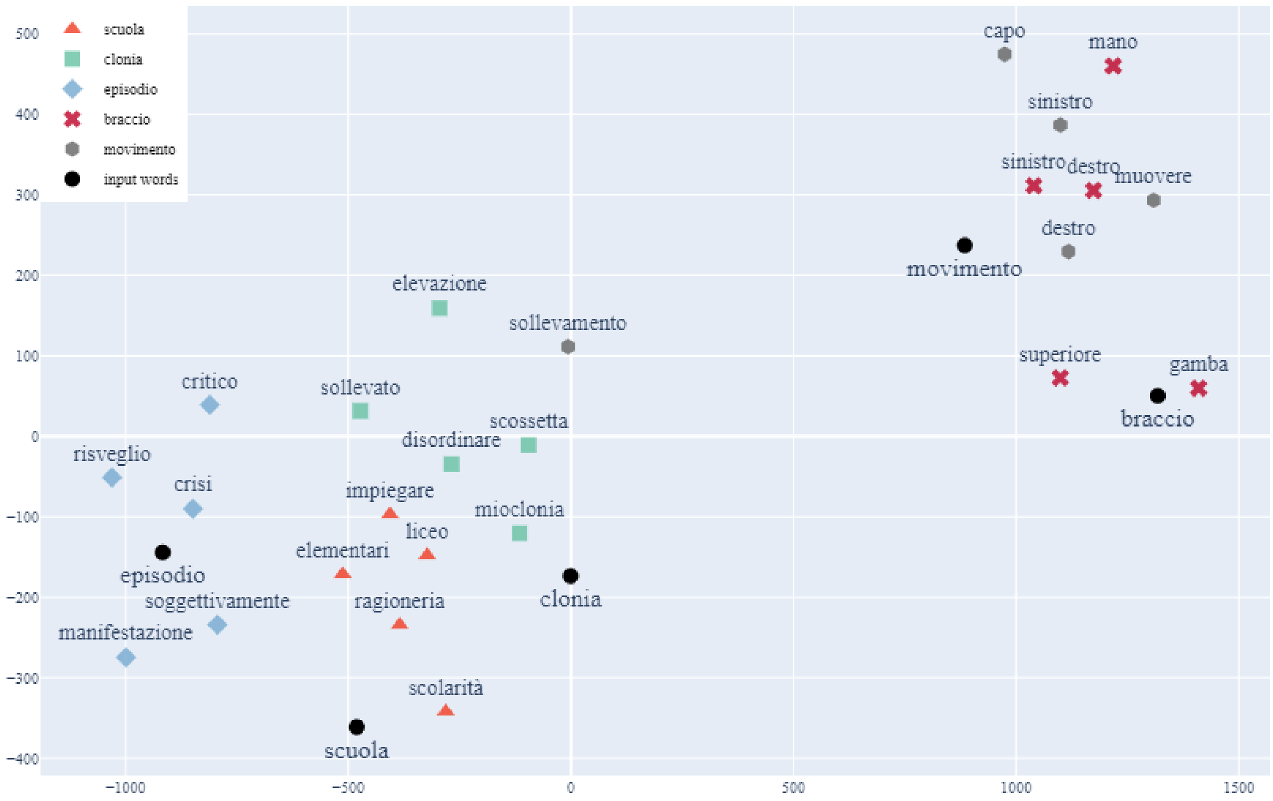

Figure S1. **Examples of clusters of Word Embedding, visualized in a two-dimensional space using *T-distributed Stochastic Neighbor Embedding* (T-SNE).** Each cluster is composed of one input word and the top 5 most similar words. Two main clusters can be distinguished in the figure, words in the upper-right part of the figure are linked to the seizure descriptions ("braccio *(arm)*" and "movimento *(movement)*") and words in the lower-left part of the figure are linked to anamnestic information ("episodio *(episode)*", "scuola *(school)*" and "clonia *(jerk)*", which is mentioned most of the times in that section).

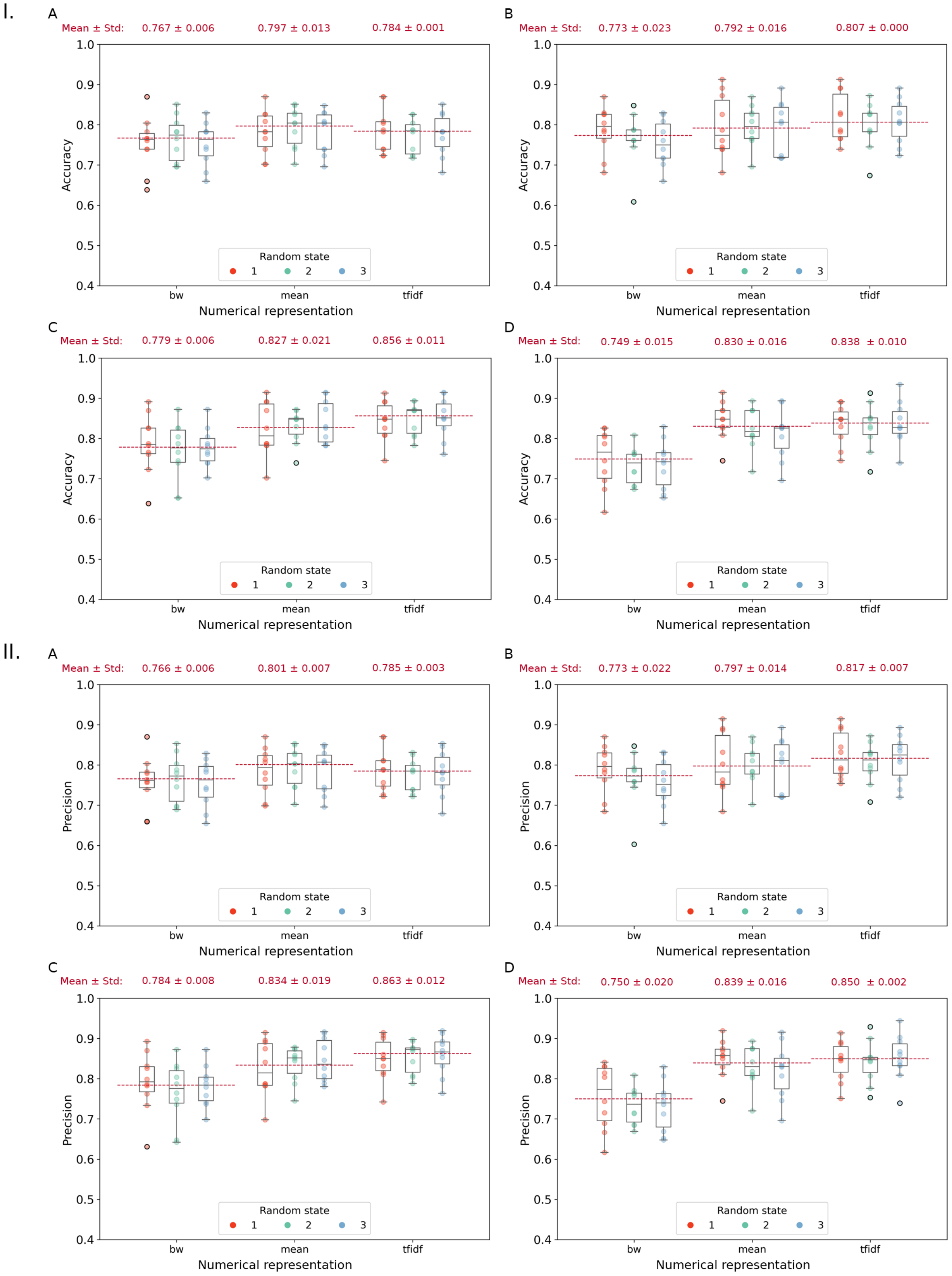
I.
A
Mean ± Std: 0.767 ± 0.006 0.797 ± 0.013 0.784 ± 0.001
Accuracy
Random state 1 2 3
bw mean tfidf
Numerical representation
B
Mean ± Std: 0.773 ± 0.023 0.792 ± 0.016 0.807 ± 0.000
Accuracy
Random state 1 2 3
bw mean tfidf
Numerical representation
C
Mean ± Std: 0.779 ± 0.006 0.827 ± 0.021 0.856 ± 0.011
Accuracy
Random state 1 2 3
bw mean tfidf
Numerical representation
D
Mean ± Std: 0.749 ± 0.015 0.830 ± 0.016 0.838 ± 0.010
Accuracy
Random state 1 2 3
bw mean tfidf
Numerical representation
II.
A
Mean ± Std: 0.766 ± 0.006 0.801 ± 0.007 0.785 ± 0.003
Precision
Random state 1 2 3
bw mean tfidf
Numerical representation
B
Mean ± Std: 0.773 ± 0.022 0.797 ± 0.014 0.817 ± 0.007
Precision
Random state 1 2 3
bw mean tfidf
Numerical representation
C
Mean ± Std: 0.784 ± 0.008 0.834 ± 0.019 0.863 ± 0.012
Precision
Random state 1 2 3
bw mean tfidf
Numerical representation
D
Mean ± Std: 0.750 ± 0.020 0.839 ± 0.016 0.850 ± 0.002
Precision
Random state 1 2 3
bw mean tfidf
Numerical representation

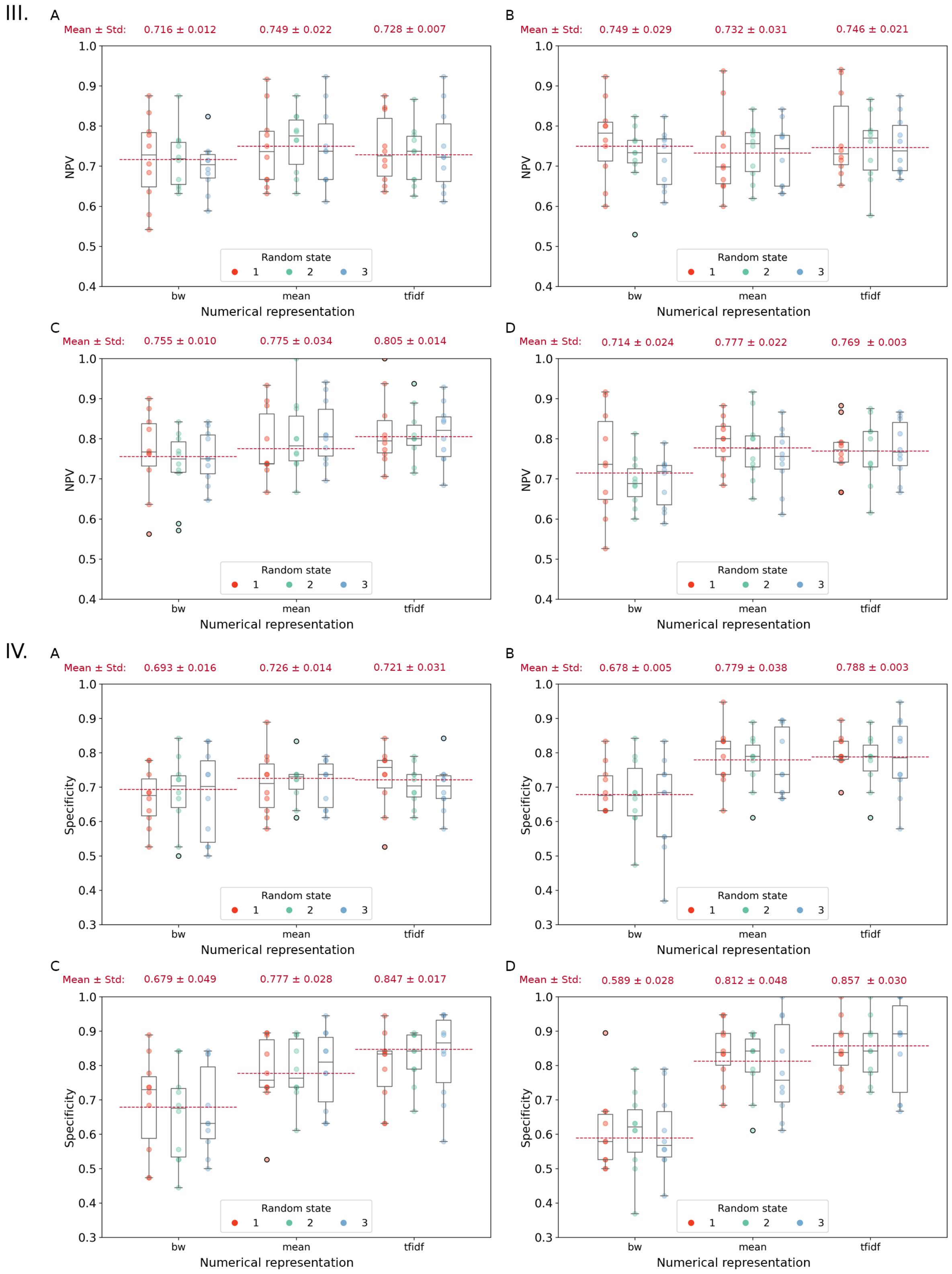

Figure S2. Model performances for the localization task on validation set in terms of (I) accuracy (which equals the weighted recall, *i.e.* sensitivity), (II) precision, (III) NPV, (IV) specificity of (A) Sparse Logistic Regression, (B) SVM with linear kernel, (C) SVM with rbf kernel, and (D) SVM with poly kernel over the three fixed random states (red, light green, and light blue) and the three numerical representations (*bw*, *mean*, and *tfidf*). For each representation and random state, the weighted F1-score values of the K-folds are showed. The red dotted lines identify the mean of second quartiles over the three random states. Numbers at the top of each panel represent $\mu \pm \sigma$ of the second quartiles over the three random states.

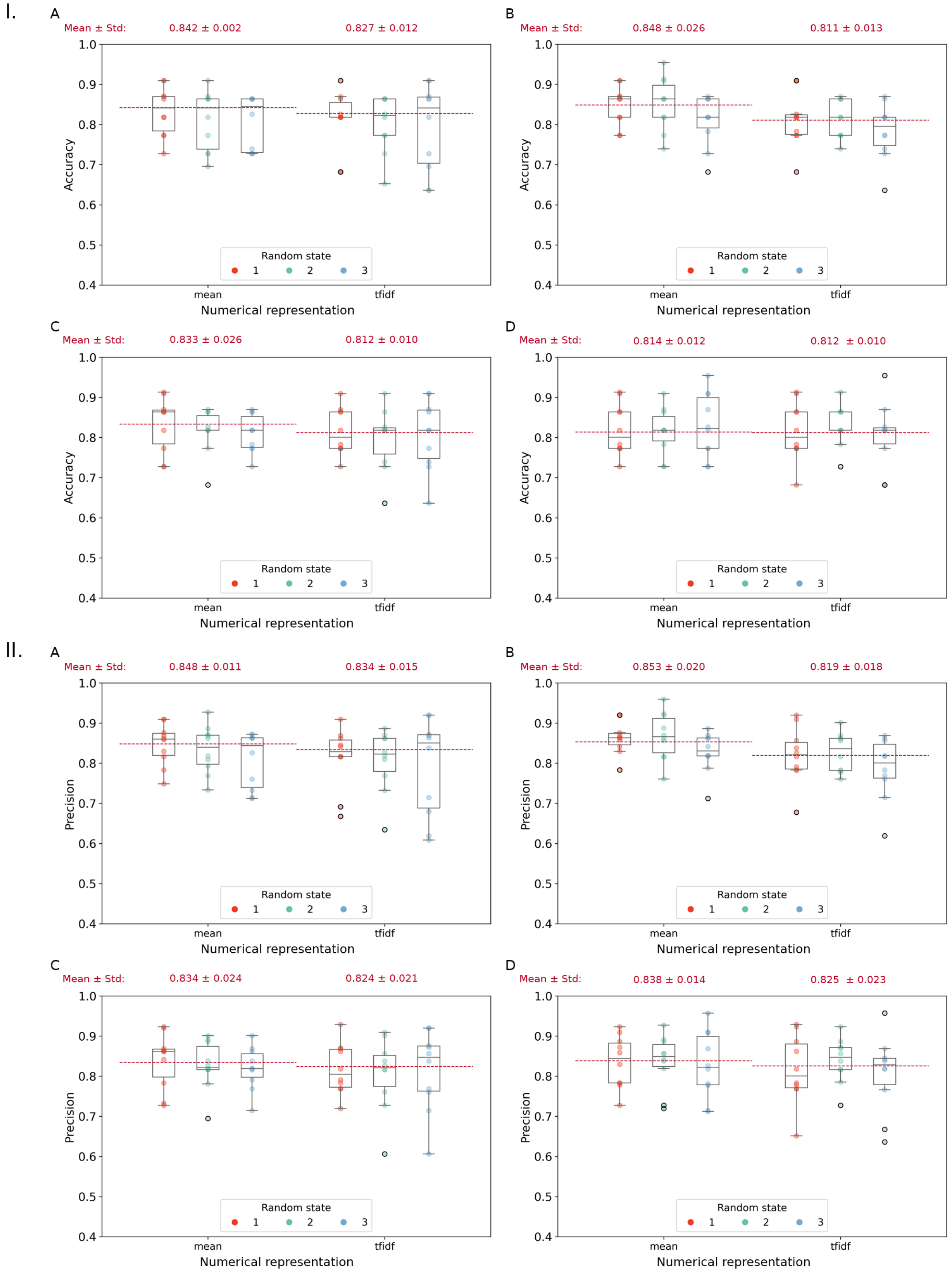
I.
A
Mean ± Std: 0.842 ± 0.002 0.827 ± 0.012
B
Mean ± Std: 0.848 ± 0.026 0.811 ± 0.013
C
Mean ± Std: 0.833 ± 0.026 0.812 ± 0.010
D
Mean ± Std: 0.814 ± 0.012 0.812 ± 0.010
Accuracy
II.
A
Mean ± Std: 0.848 ± 0.011 0.834 ± 0.015
B
Mean ± Std: 0.853 ± 0.020 0.819 ± 0.018
C
Mean ± Std: 0.834 ± 0.024 0.824 ± 0.021
D
Mean ± Std: 0.838 ± 0.014 0.825 ± 0.023
Precision
Random state
1 2 3
mean
tfidf
Numerical representation
1.0
0.9
0.8
0.7
0.6
0.5
0.4

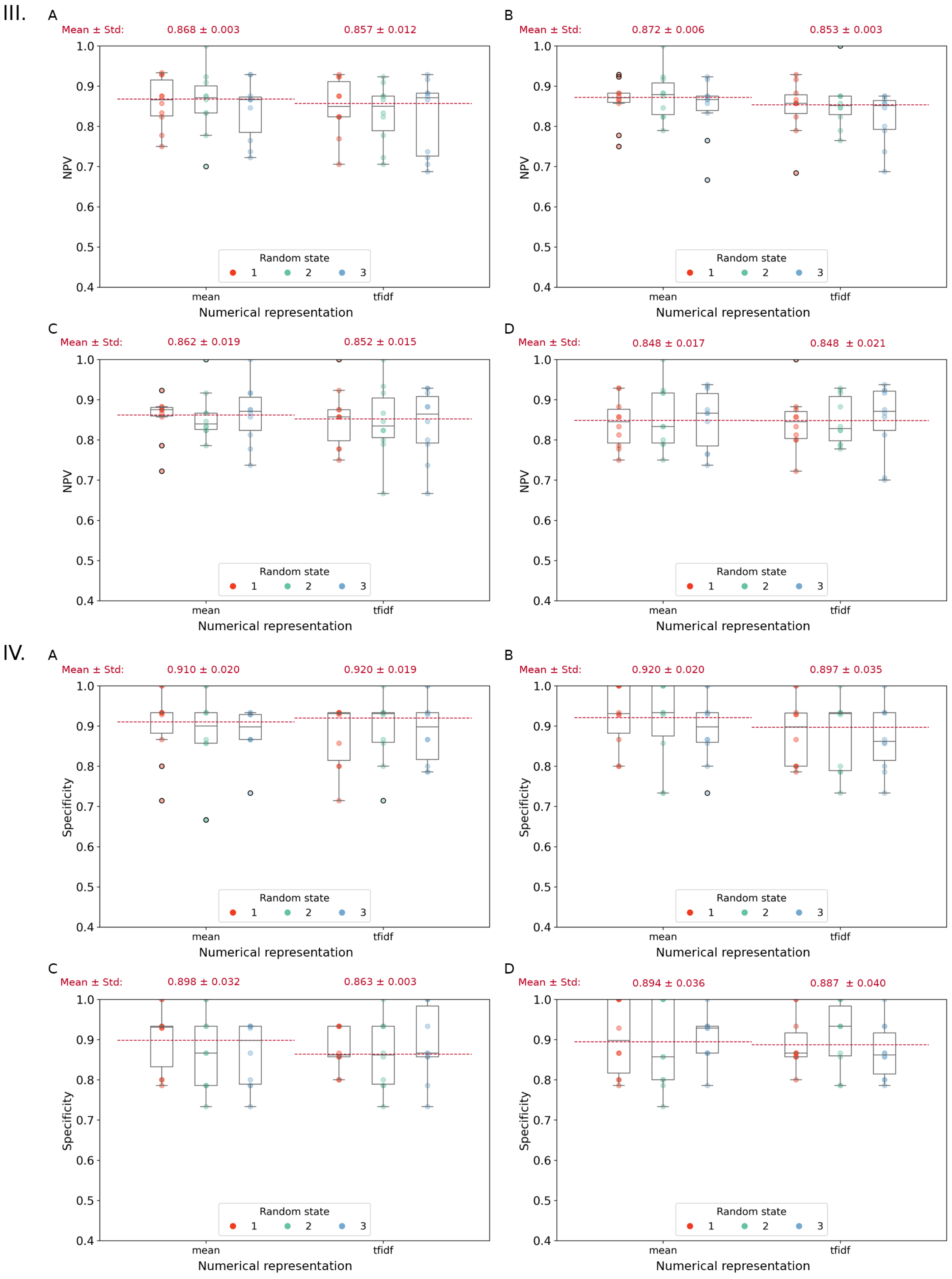

Figure S3. Model performances for the localization in Frontal/Posterior region task on validation set in terms of (I) accuracy (which equals the weighted recall, *i.e.* sensitivity), (II) precision, (III) NPV, (IV) specificity of (A) Sparse Logistic Regression, (B) SVM with linear kernel, (C) SVM with rbf kernel, and (D) SVM with poly kernel over the three fixed random states (red, light green, and light blue) and the three numerical representations (*bw*, *mean*, and *tfidf*). For each representation and random state, the weighted F1-score values of the K-folds are showed. The red dotted lines identify the mean of second quartiles over the three random states. Numbers at the top of each panel represent $\mu \pm \sigma$ of the second quartiles over the three random states.

I.

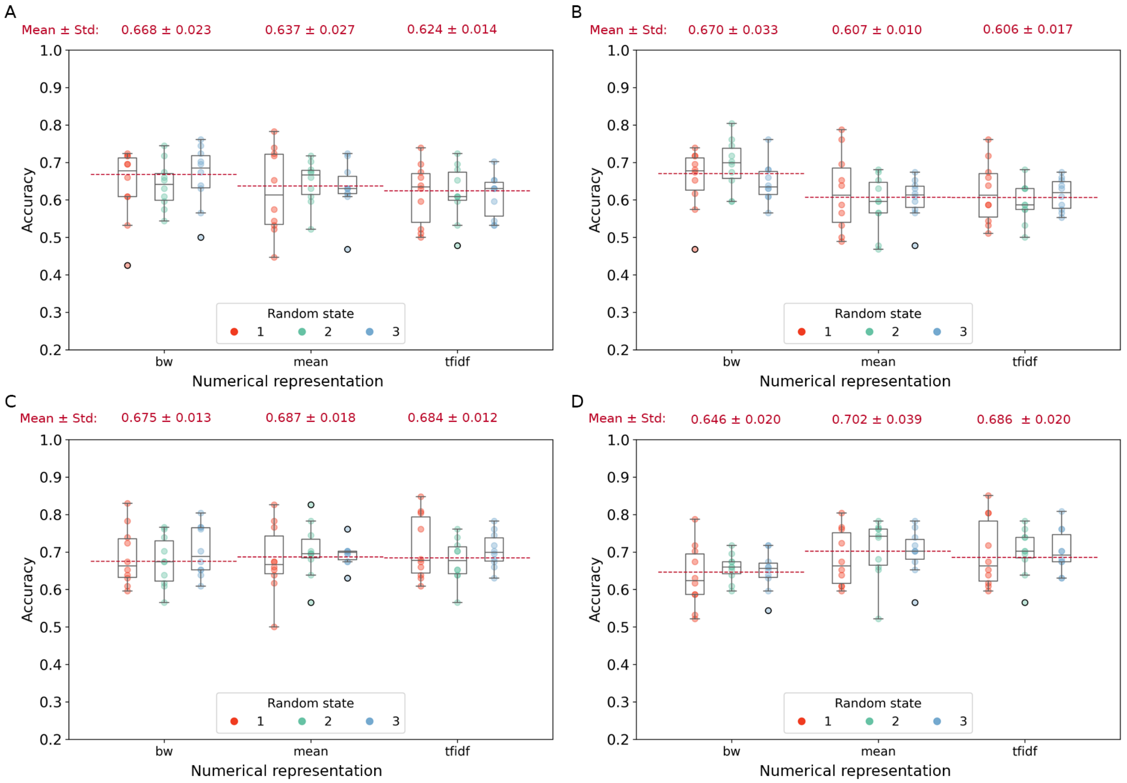
A
Mean ± Std: 0.668 ± 0.023 0.637 ± 0.027 0.624 ± 0.014
Accuracy
Random state 1 2 3
bw mean tfidf
Numerical representation
B
Mean ± Std: 0.670 ± 0.033 0.607 ± 0.010 0.606 ± 0.017
Accuracy
Random state 1 2 3
bw mean tfidf
Numerical representation
C
Mean ± Std: 0.675 ± 0.013 0.687 ± 0.018 0.684 ± 0.012
Accuracy
Random state 1 2 3
bw mean tfidf
Numerical representation
D
Mean ± Std: 0.646 ± 0.020 0.702 ± 0.039 0.686 ± 0.020
Accuracy
Random state 1 2 3
bw mean tfidf
Numerical representation

II.

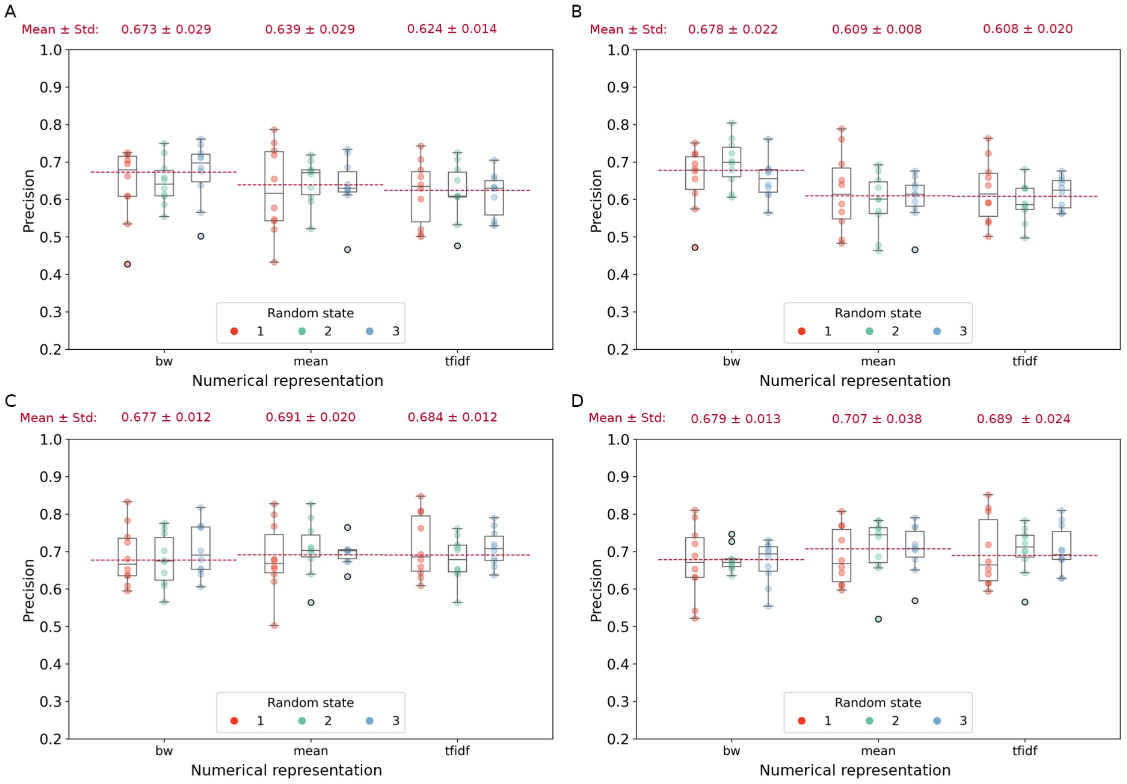
A
Mean ± Std: 0.673 ± 0.029 0.639 ± 0.029 0.624 ± 0.014
Precision
Random state 1 2 3
bw mean tfidf
Numerical representation
B
Mean ± Std: 0.678 ± 0.022 0.609 ± 0.008 0.608 ± 0.020
Precision
Random state 1 2 3
bw mean tfidf
Numerical representation
C
Mean ± Std: 0.677 ± 0.012 0.691 ± 0.020 0.684 ± 0.012
Precision
Random state 1 2 3
bw mean tfidf
Numerical representation
D
Mean ± Std: 0.679 ± 0.013 0.707 ± 0.038 0.689 ± 0.024
Precision
Random state 1 2 3
bw mean tfidf
Numerical representation

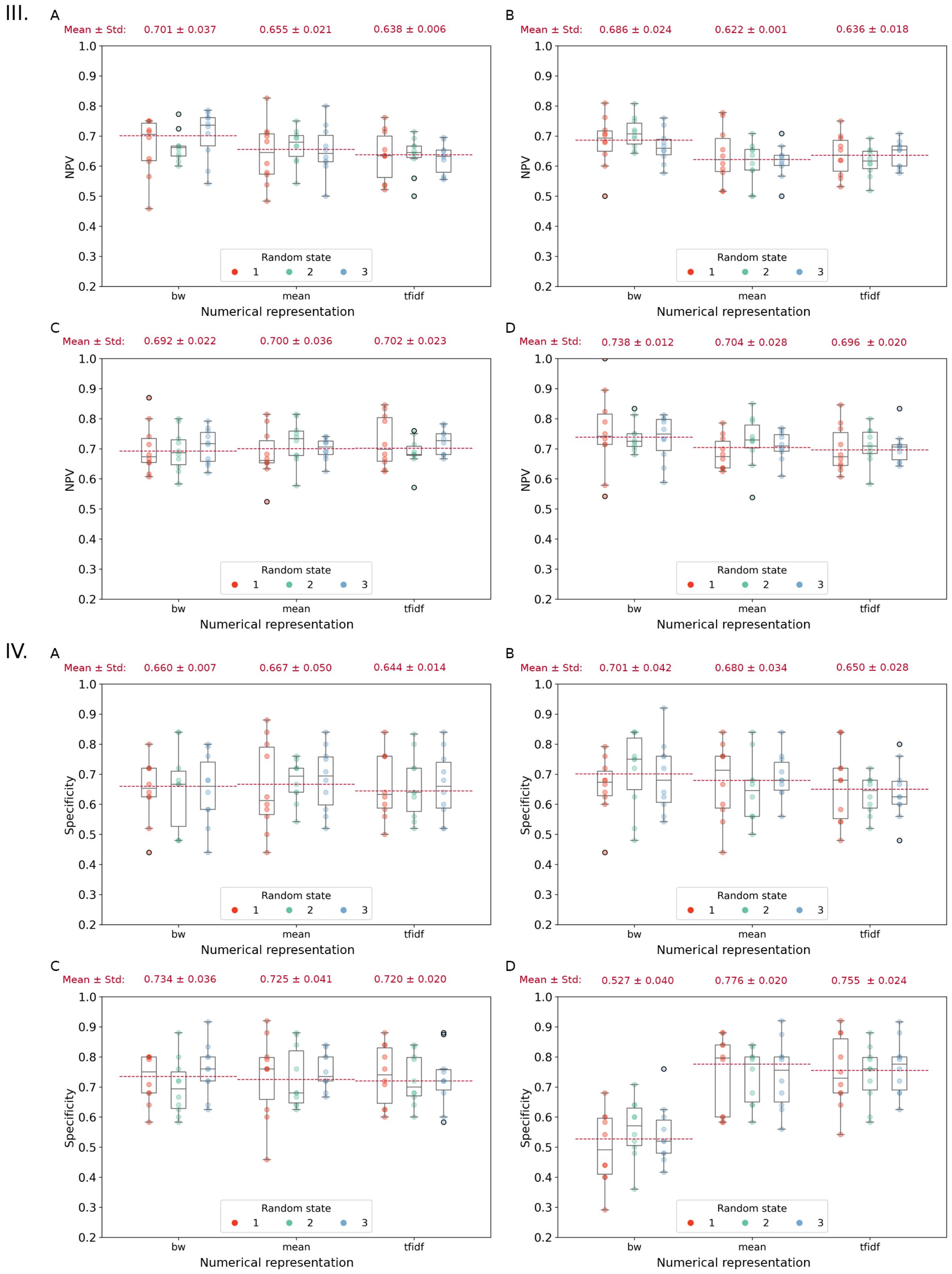

Figure S4. Model performances for the lateralization task on validation set in terms of (I) accuracy (which equals the weighted recall, *i.e.* sensitivity), (II) precision, (III) NPV, (IV) specificity of (A) Sparse Logistic Regression, (B) SVM with linear kernel, (C) SVM with rbf kernel, and (D) SVM with poly kernel over the three fixed random states (red, light green, and light blue) and the three numerical representations (*bw*, *mean*, and *tfidf*). For each representation and random state, the weighted F1-score values of the K-folds are showed. The red dotted lines identify the mean of second quartiles over the three random states. Numbers at the top of each panel represent $\mu \pm \sigma$ of the second quartiles over the three random states.
